# Food for thought: how nutrients regulate root system architecture

**DOI:** 10.1016/j.pbi.2017.06.008

**Published:** 2017-10

**Authors:** Zaigham Shahzad, Anna Amtmann

**Affiliations:** Institute of Molecular, Cellular and Systems Biology, College of Medical, Veterinary, and Life Sciences, University of Glasgow, Glasgow G12 8QQ, United Kingdom

## Abstract

The spatial arrangement of the plant root system (root system architecture, RSA) is very sensitive to edaphic and endogenous signals that report on the nutrient status of soil and plant. Signalling pathways underpinning RSA responses to individual nutrients, particularly nitrate and phosphate, have been unravelled. Researchers have now started to investigate interactive effects between two or more nutrients on RSA. Several proteins enabling crosstalk between signalling pathways have recently been identified. RSA is potentially an important trait for sustainable and/or marginal agriculture. It is generally assumed that RSA responses are adaptive and optimise nutrient uptake in a given environment, but hard evidence for this paradigm is still sparse. Here we summarize recent advances made in these areas of research.

## Highlights

• Root system architecture is a precise readout of nutrient signalling.
• RSA is regulated by transcriptional, translational, redox and cell-wall processes.
• Peptides act as local and systemic signals in N and P signalling.
• Crosstalk of nutrient signalling pathways underpins interactive effects.
• RSA correlates with nutrient use efficiency and yield in crop genotypes.

**Current Opinion in Plant Biology** 2017, **39**:80–87

This review comes from a themed issue on **Cell signalling and gene regulation**

Edited by **Tzyy-Jen Chiou**, **Wayne K Versaw** and **Toru Fujiwara**

For a complete overview see the Issue and the Editorial

Available online 30th June 2017

**http://dx.doi.org/10.1016/j.pbi.2017.06.008**

1369-5266/© 2017 The Authors. Published by Elsevier Ltd. This is an open access article under the CC BY license (http://creativecommons.org/licenses/by/4.0/).

## Introduction

Roots are pivotal for anchorage of land plants in the soil and for efficient uptake of water and mineral nutrients, thus playing a crucial role in plant fitness. The root system is composed of embryonic roots (primary root in Arabidopsis, primary and seminal roots in a few cereal crops) and postembryonic roots (lateral roots in Arabidopsis, lateral, brace and crown roots in cereals). Root system architecture (RSA), the overall spatial arrangement of individual parts of the root system, is an important factor determining how efficiently plants can access resources. RSA is highly plastic both genetically and environmentally. Thus, different species or ecotypes have evolved different RSAs depending on the prevailing soil conditions. Considerably different RSAs can also be adopted within the same genotype and even within the life span of a single plant because the rate at which individual parts of the root system develop and grow can be altered by short-term environmental signals, including changes in water, nutrient and oxygen availability or pathogens and pests. Unlike animals, plants cannot move away from unfavourable sites. Hence, plant responses to fluctuating soil conditions are based on altered growth and development, and they require sophisticated sensing and signalling mechanisms. While the mechanisms controlling RSA responses to individual nutrients, especially nitrogen (N) and phosphorus (P), have been extensively investigated, the crosstalk between different nutrient signals and the benefits of RSA responses in a particular condition are yet to be characterized systematically. In this review, we will highlight some of the recently identified molecular mechanisms that underpin RSA responses to single and combined nutrient stress, and we will explore the potential benefits of RSA responses for plant performance.

## The genetic and environmental context of RSA responses

**Figure 1:**
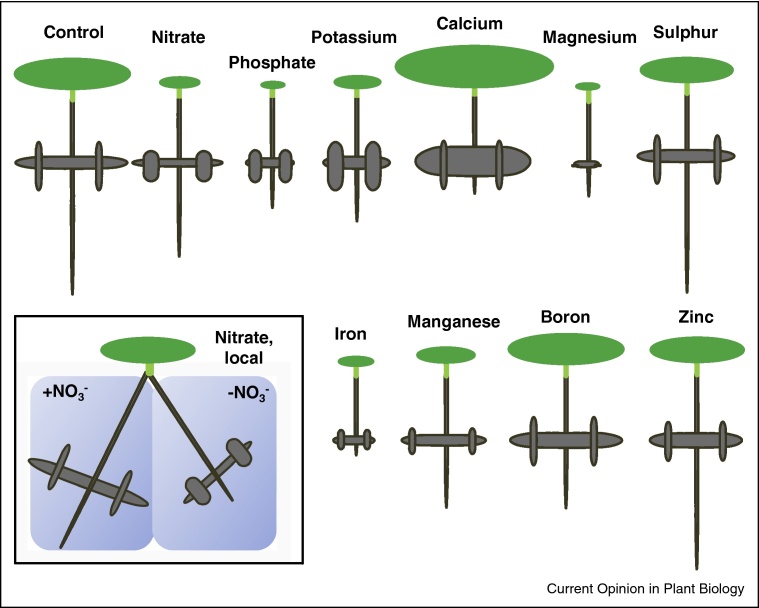
Schematic phenotypes of *Arabidopsis thaliana* Col-0 plants in single-nutrient deficiency. Representative phenotypes are shown for plants grown on vertical agar plates with media lacking the specified nutrient (except for nitrate which was supplied at low level), based on published data [1•, 2•]. Phenotypes in nutrient-sufficient media and phenotypes reported for localized nitrate treatment [3] are shown for comparison. Length of primary and lateral roots is represented by the length of the grey spheres, and lateral root density is represented by their width. Green ovals represent shoot biomass.

Most studies into nutrient signalling and RSA have been carried out in *Arabidopsis thaliana* (Col-0) grown on agar plates, and before we review the knowledge generated by these studies, it is important to discuss their transferability to other species and root environments. The endogenous developmental programmes underpinning RSA differ between plant species (especially between monocots and dicots) which is apparent in different RSAs under identical nutrient sufficient conditions. Therefore, a RSA phenotype produced in response to a particular nutritional stimulus can differ between species even if the signalling pathway feeding into the different developmental programmes is conserved. Furthermore, the phenotypic consequence of a change in nutrient supply in a given genotype will depend on exact nutrient concentration, nutrient distribution and gradients, concentrations of other nutrients, developmental stage of the plant, and any factors that determine plant growth rate and nutrient demand, for example, light, humidity, etc. [1•, 2•, 3]. Differences in these factors are likely to explain many of the discrepancies between studies carried out with the same genotype, for example, *A. thaliana* Col-0. Clearly one has to be careful when drawing wider conclusions from observations made in a specific genotype or environmental condition. However, this does not imply that meaningful data can only be derived from field-based studies on crops. Ultimately, whether findings can be translated between species and environments or not depends on the question posed. If the RSA itself is the focus of interest (e.g. as a trait to enhance agricultural yield or as a phenotype to solve specific developmental questions) the precise experimental context is of essence and findings may not be transferable to other genotypes or environments. If, however, RSA responses are used as readout to enable the identification of nutrient sensors and signalling components, the context is less crucial, and artificial conditions are often more informative than natural conditions. Figure 1 exemplifies the usefulness of Arabidopsis RSA developing on an agar surface for reporting the action of nutrient-specific sensing and signalling pathways. Each specific nutrient treatment leads to a distinct multifactorial phenotypic output that can be interrogated through genetic, pharmacological and physiological manipulations. In the following, we will describe the latest insights that have been obtained using this latter approach.

## Root system architecture responses to individual nutrients

### Nitrate

In Arabidopsis plants grown with uniform nutrient supply, the dose–effect curve for nitrate and lateral root (LR) length is bell-shaped, whereas primary root (PR) length is continuously inhibited by lowering nitrate over the same concentration range [4]. The effect of nitrate on LR initiation is controversial; several studies report a positive effect of nitrate on LR density [5, 6] while others have found no effects of nitrate on LR number or density [7]. Non-uniform, localized nitrate supply stimulates LR elongation in nitrate rich patches [3].

**Figure 2:**
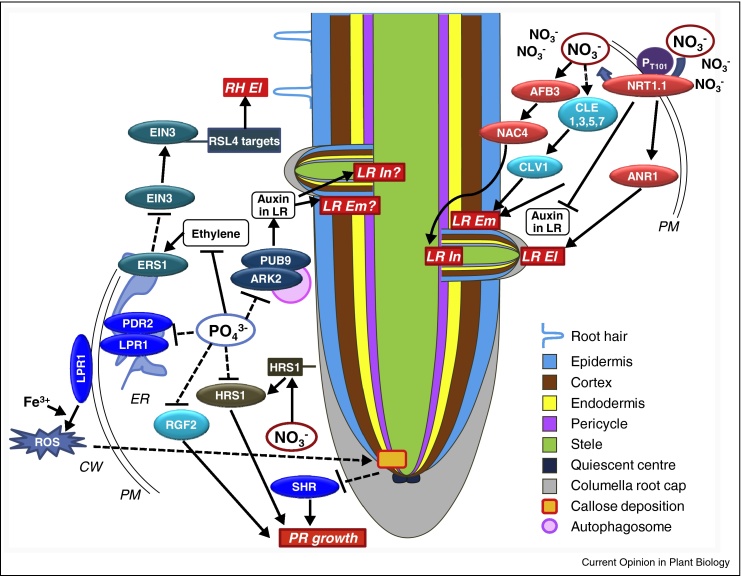
Summary of signalling pathways underpinning root system architecture responses to nitrate or phosphate deficiency. Components of signalling pathways discussed in this review are shown as coloured boxes (genes) or circles (proteins). Arrows and bars indicate positive and negative regulation, respectively. Broken lines represent indirect pathways or circumstantial evidence. RSA targets (red boxes) are primary root (*PR*) growth, lateral root initiation (*LR In*), emergence (*LR Em*) and elongation (*LR El*), as well as root hair elongation (*RH El*). Cellular localization is indicated as cell wall (*CW*), plasma membrane (*PM*) or endoplasmic reticulum (*ER*). Root tissues and other features are coloured as detailed in the legend.

NITRATE TRANSPORTER 1.1, NRT1.1 (NPF6.3), is a plasma membrane nitrate transporter that alters its affinity depending on nitrate availability, and functions as a nitrate sensor upstream of transcriptional low-nitrate responses [8]. It also acts as a basipetal auxin transporter in the developing LR tips thereby controlling both local and systemic LR responses to low nitrate [3, 9]. Phosphorylation of NRT1.1 at T101 is important for auxin transport activity and for repression of LR emergence in low nitrate (Figure 2) [10^•^]. A recent study [11] resolved the longstanding question how such a function is compatible with the observed transcriptional down-regulation of NRT1.1 under low nitrate, both at the whole root level and in LR primordia [3, 12]. Bouguyon and colleagues showed that in contrast to NRT1.1-mRNA the NRT1.1-protein levels are increased in LR primordia of nitrate starved roots with a concomitant decrease of auxin accumulation in LR primordia. These findings affirm a role of NRT1.1 in the low-nitrate RSA response, and the challenge now is to identify the mechanism regulating NRT1.1 protein stability. In contrast to the well documented stimulatory effects of localized nitrate on LR growth, a recent study found an increase in LR length under uniform low-nitrate conditions, which was dependent on the auxin biosynthesis enzyme TRYPTOPHAN AMINOTRANSFERASE RELATED 2, TAR2 [13]. Nitrate was supplied in the form of NH_4_NO_3_ in contrast to previous studies where it was supplied as KNO_3_, thus making it difficult to distinguish between the effects of NH_4_ and NO_3_.

One group of downstream targets of NRT1.1 mediated N-signalling are ANR1 MADS-box transcription factors, which positively regulate LR elongation in response to localized nitrate supply [14]. In a uniform low-nitrate environment Arabidopsis plants overexpressing ANR1 exhibit longer LRs while no alteration in LR density and PR length was observed compared to wild type [15]. Rice harbours five ANR1-like MADS transcription factors, four of which (OsMADS 23, 25, 27, 57 and 61) are targets of a microRNA, miR144-a, which is conserved in monocots and absent in Arabidopsis [16]. The overexpression of miR144-a in rice reduces the expression of these MADS-box transcription factors and abolishes LR stimulation by localized nitrate treatment, suggesting that one or more of these rice ANR1-like genes play a similar role to that of Arabidopsis ANR1. A rice homologue of Arabidopsis NRT1.1, NRT1.1B, was recently identified by QTL mapping of natural genetic variation of N signalling and N use efficiency [17^•^]. However, a comprehensive characterization of the role of rice NRT1.1B in controlling root developmental responses to nitrate is still missing. Furthermore, it is also not known if NRT1.1B acts upstream of rice ANR1-like genes to regulate their expression in addition to the miR144-a.

An essential role of small peptides in perception of N and alteration of root development was recently discovered in Arabidopsis. In a heterogeneous nitrate environment, C-terminally encoded peptides (CEPs) are secreted by the N-starved roots and are considered to be translocated to the shoot where they are perceived by two leucine-rich repeat receptor kinases (CEPR1, CEPR2 LRK-RKs). This process triggers systemic N-demand signalling in roots facing nitrate rich patches as evidenced by analysing the mRNA regulation of *NRT* genes [18]. In addition to CEP peptides, CLE (CLAVATA3/Endosperm surrounding region-related) peptides were also detected in the xylem sap [19], and this signal also appears to be involved in the adjustment of root development to N-need (Figure 2) [4]. mRNAs of CLE peptides are induced by low nitrate and repressed by high nitrate, and overexpression of Arabidopsis CLE1 to 7 was shown to inhibit LR development under uniform low nitrate conditions [4]. In summary, there is increasing evidence for the involvement of peptides in N signalling, but the integration of these signalling pathways with the NRT1.1-dependent signalling pathway remains to be resolved.

A detailed discussion of how nitrate modulates LR initiation and PR growth can be found elsewhere [20]. Briefly, the current state of knowledge suggests that LR initiation is regulated by an interaction between nitrate and auxin signalling pathways and involves NRT1.1-dependent nitrate accumulation that induces the AUXIN SIGNALLING F-BOX 3, AFB3, and a NAC domain containing transcription factor, NAC4, acting downstream of AFB3 (Figure 2) [6, 21].

### Phosphate

Phosphate starvation strongly inhibits PR, stimulates LR growth, and enhances root hair production. Numerous genetic components regulating root developmental responses to phosphate starvation have been identified [22]. In Arabidopsis Col-0 LOW PHOSPHATE ROOT 1 (LPR1) and PHOSPHATE DEFICIENCY RESPONSE 2 (PDR2) appear to be central for sensing phosphate deprivation and triggering PR growth inhibition (Figure 2) [23]. LPR1, and its homologue LPR2, encode ferroxidases while PDR2 encodes a P5-type ATPase of unknown substrate specificity. The LPR1 and PDR2 proteins are expressed in the same domains of the root apical meristem (RAM) [24]. Phosphate starvation-induced PR growth inhibition is markedly reduced in *lpr1* and *lpr2* single mutants and the response is completely abolished in *lpr1lpr2* double mutant plants. By contrast, *pdr2* mutants exhibit a hypersensitive short-root phenotype under phosphate limitation [25, 26••]. Phosphate deprived wild type PRs show increased radial cell division in the root meristem and disorganization of the quiescent centre [27^•^]. Phosphate deficiency also leads to apoplastic callose deposition possibly due to enhanced ROS production in stem cell niche and RAM (Figure 2), which is more pronounced in *pdr2* mutant and not evident in *lpr1lpr2* mutant plants [26^••^]. Deposition of callose interferes with the cell to cell movement of SHORT ROOT HAIR (SHR), a key transcription factor regulating radial root patterning, which could explain the impact on RAM maintenance. The *lpr1lpr2pdr2* triple mutant plants do not show callose deposition or hypersensitive short-root phenotype under phosphate deprivation which indicates that LPR1 works downstream of PDR2 in the same pathway [24, 26••]. Recent studies highlight that the impairment of root meristematic activity under phosphate deficient conditions is preceded by an impairment of cell expansion in the root elongation zone, which is again LPR1 dependent. A SENSITIVE TO PROTON TOXICITY1-ALUMINUM-ACTIVATED MALATE TRANSPORTER1 (STOP1-ALMT1) module acts upstream of LPR1 to control this early response [28, 29].

A role of peptide signalling mechanisms is also emerging for low-P sensing and modulation of RSA (Figure 2). Arabidopsis mutant plants for RGF2, a member of the ROOT GROWTH FACTOR/GOLVEN/CLE-like (RGF/GLV/CLEL) family of peptides, show a hypersensitive short-root phenotype after transfer to low-P media [27^•^], indicating that RGF2 maintains longitudinal PR growth under phosphate deprivation. Mutant analysis further revealed that RGF2 also enhances radial divisions in epidermis, cortex, and endodermis. By contrast, another RGF gene, RGF1, represses radial divisions in these tissues [27^•^]. This suggests that the different RGF peptides play specific roles to modulate RSA under P deficiency. Whether and how a peptide signalling pathway is integrated with the PDR2-LPR1 module remains to be investigated.

A functional module comprising S-DOMAIN RECEPTOR KINASE 1-6 (SDK6 (ARK2)) and its interacting partner, the U-box/ARM REPEAT containing E3 ligase AtPUB9, plays a role in LR development in phosphate deprived roots [30]. An Arabidopsis *ark2pub9* double mutant exhibits severe reduction of phosphate starvation-induced LR growth stimulation, which is associated with decreased auxin accumulation in PR as well as LR tips compared to wild type (Figure 2). The PUB9 protein is localized to the autophagosomes in the presence of ARK2 indicating a potential role of autophagy in stimulating LR formation under phosphate deficient conditions [30]. This notion was further supported by the lack of stimulation of LR formation when P-starved roots were co-treated with 3-methyladenine (3-MA), an autophagy inhibitor.

As mentioned above, phosphate starvation also induces root hair production. The molecular components of this response were recently identified through a large-scale genetic screen. A dominant negative mutation in the ethylene receptor ERS1, ‘hypersensitive to phosphate starvation 5’ (*hsp5*), resulted in a constitutive increase in the number and length of root hairs, and the phenotype was markedly pronounced under phosphate deficient conditions [31]. This mutation causes a constitutive ethylene response due to enhanced protein accumulation of ETHYLENE INSENSITIVE 3 (EIN3), a key transcription factor involved in the ethylene signalling pathway (Figure 2). Interestingly, EIN3 protein, which was also found to be induced by phosphate deprivation, binds to the direct targets of ROOT HAIR DEFECTIVE 6-like 4 (RSL4), a transcription factor controlling root hair development, thus triggering the signalling pathway regulating root hair development.

### Other nutrients

Although root system architectural responses to deficiency of other nutrients than N and P have been reported [1•, 2•, 30], very little is known about the mechanisms controlling them. In general, at the morphological level, inhibition of PR growth is a common response to most nutrient deficiencies, except for sulphur and zinc. However, the severity of inhibition varies between nutrients. Amongst the macronutrients phosphate is by far the most important nutrient determining PR length in Arabidopsis Col-0 [1^•^], but in some other accessions potassium (K) has a stronger impact than P [32]. Deficiency-induced LR responses vary considerably between nutrients producing nutrient-specific patterns of LR length, density and branching [2^•^]. In Col-0, K starvation leads to a strong inhibition of first-order LR length while branching is stimulated, leading to increased second-order LR density. The branching response is compromised in knockout mutants of AKT1, an inward rectifying K-channel, CIPK23, a CBL-interacting protein kinase, and NRT1.1 [1^•^]. CIPK23 when bound to CBL1/9 phosphorylates AKT1 and enhances channel activity [33]. NRT1.1 is also a target of CIPK23 [8], which might explain its involvement in the K-starvation response. However, AKT1 is not the main pathway for K-uptake under K-starvation, suggesting that its primary role in RSA regulation is that of a K-sensor (‘transporter-receptor’), similar to the role of NRT1.1 in N-signalling. This view is further supported by the finding that the high-affinity K-uptake transporter HAK5, which is crucial for K-nutrition in low K, is not required for the RSA response [1^•^].

Several other ions including micronutrients also modulate LR features (Figure 1) [2^•^]. Starvation for calcium or boron increases first-order LR density without significantly affecting LR length. Magnesium or iron starvation result in a reduction of first-order LR density and length. Manganese starvation inhibits LR length and slightly reduces branching whereas zinc deficiency also inhibits LR growth but stimulates LR branching. In summary, plants respond to deficiencies of most nutrients with nutrient-specific RSA phenotypes. The next challenge is now to extend our knowledge on signalling pathways controlling RSA to nutrients other than P and N.

## Responses to combined nutrient stress

Whether plants grow in the wild or in agriculture, they will often face multiple nutritional deficiencies. The importance of interactive effects of nutrients on RSA has only recently been highlighted in a study applying multiple combinations of sufficient and low N, P, K and S, as well as high and low light, to Arabidopsis plants [1^•^]. Various types of antagonistic, synergistic, or null effects of combined nutrient deficiencies as compared to single nutrient deficiencies were observed. For instance, S was found to have interactive effects on RSA with several other nutrients despite not causing any RSA phenotypes in singe deficiency, while K and N increased LR branching in single but not in double deficiency [1•, 2•]. Clearly, conventional single-nutrient deficiency experiments can highlight only a very small part of the complex nutrient signalling network that underpins RSA in different soil conditions. Large-scale experiments would need to be carried out, using a multi-dimensional design to enable quantification of RSA in various concentration ratios of all essential nutrients.

At least for a subset of combined nutritional stresses perception and signalling crosstalk has recently been studied. A molecular mechanism integrating phosphate and nitrate signals was described for Arabidopsis Col-0 [34^••^]. A major part of the phosphate starvation-induced PR inhibition was found to be controlled by the transcription factor HYPERSENSITIVE TO LOW Pi-ELICITED PR SHORTENING (HRS1) and its homologue HHO1, but only when nitrate was present in the growth media [34^••^]. HRS1 and HHO1 are transcriptionally induced early during nitrate starvation in an NRT1.1-dependent manner, but HRS1 protein is stabilized by phosphate starvation (Figure 2). This indicates that transcriptional and post-transcriptional regulation of HRS1 by nitrate and phosphate respectively constitutes a mechanism to exhibit an integrated root response to two distinct nutritional signals.

Cross-talk between phosphate and heavy metal nutrients such as iron and zinc has long been appreciated. In particular, the phosphate starvation-induced inhibition of PR growth has been shown to be dependent on the availability of iron [35], which is likely to be due to the importance of Fe^3+^ in the PDR2-LPR1 signalling module [26^••^]. LPR1 protein exhibits dual sub-cellular localization in the endoplasmic reticulum and in the cell wall, but is considered to function primarily as a cell wall ferroxidase. LPR1-dependent apoplastic Fe^3+^ accumulation in the root tip is potentially a source of ROS production. In the latest model, ROS accumulation then leads to meristem-specific callose formation and restriction of cell to cell movement of SHR1, which ultimately impedes PR development (Figure 2, and above).

## Benefits of adjusting RSA to nutrient availability

RSA plasticity under nutrient stress is generally described as an adaptive measure to enhance nutrient uptake through improved soil foraging [36]. However, there is surprisingly little experimental evidence to prove this concept. Intra-species variation of RSA provides an excellent opportunity to assess the consequences of different root architectures on nutrient uptake efficiency, shoot growth and yield in different conditions in a similar genetic background.

In the case of N, a steep, cheap and deep root system has been proposed to be ideal for N acquisition, because nitrate is very mobile in the soil [37]. This root model was indeed validated by studying various genotypes of maize. Maize ‘FL’ genotypes with few but long LRs performed better under low-N than ‘MS’ genotypes with many but short LRs [37, 38•]. LR density was found to be negatively correlated with the root depth which was positively correlated with plant N content, shoot growth and grain yield under low N conditions. One possible explanation is that allocation of resources required for root growth such as sugars is more efficiently relocated in FL genotypes, thus decreasing the cost of root growth while improving exploration of the soil for nitrate. Similarly, rice genotypes that showed less inhibition of root length exhibited higher nitrate uptake, nitrogen use efficiency and yield under low N conditions [39].

In the case of P deficiency, a topsoil-foraging root system is considered to be better than a deep root system as phosphate often accumulates in topsoil layers [40, 41]. Indeed, in maize genotypes higher LR branching density was correlated with higher yield on P deficient soils [42]. Evidence for the importance of total root length and root surface area for P acquisition can also be found in phenotypes related to natural and transgenic alleles of *PHOSPHORUS STARVATION TOLERANCE 1* (*PSTOL1*), a gene underlying a major QTL of P-deficiency tolerance in rice [43]. Furthermore, overexpression of expansins, for example, *GmEXPB2* in soybean or *TaEXPB23* in tobacco, increased LR number and improved phosphorus use efficiency [44, 45, 46]. Root hair elongation also contributes to P acquisition under P deficient conditions as shown for the root hair-less Arabidopsis line, NR23 [47]. Using inbred lines of common bean differing for shallow basal root angle and root hair length and density, it was found that the traits synergistically improved P acquisition under phosphate deprivation [48].

While these studies strongly support the importance of RSA for nutrient uptake and yield, they are mostly correlative and often limited by poor control of the nutrient profile in the soil and lack of knowledge on depletion kinetics in the rhizosphere. Furthermore, some of the observed differences may be based on differences in overall root size rather than differences in the spatial arrangement of individual root parts. To obtain a better understanding of the exact consequences, costs and constraints of different RSAs in different root environments it will be necessary to develop an experimental system that allows precise spatiotemporal control and monitoring of nutrient concentrations in the rhizosphere while enabling continuous measurement of RSA features and physiological parameters.

## Conclusions

Nutrient sensing and signalling continues to be an important and active field of research, and quantification of root system architecture as a phenotypic output has provided excellent opportunities to identify crucial signalling components. Over recent years novelty has particular arisen from firstly, the integration of transcriptional, translational, redox and cell-wall based regulatory processes, secondly, the discovery of peptide signals in local and systemic nutrient signalling, thirdly, the identification of molecular hubs underpinning interactive effects of different nutrients, and finally, the correlation of RSA with nutrient use efficiency and yield in crop genotypes. A concerted effort is now required to better control and monitor the nutrient profile around the root and to systematically manipulate several nutrients as well as other environmental factors that determine growth and development. Combined with genetics, particularly the exploration of natural variation, such a systemic approach could generate the necessary knowledge to generate models that can predict RSA for any genotype for any given combination of nutrients. Linking RSA to nutrient uptake, growth and yield would be the next step. It is possible that nutrient-use efficiency under a moderate fertilizer regime could be increased by de-sensitizing the plant for nutrient-deficiency signals. Exploration of this and other crop improvement strategies requires a precise understanding of how nutrient-signalling networks are hard-wired.

## References and recommended reading

Papers of particular interest, published within the period of review, have been highlighted as:

• of special interest
•• of outstanding interest
